# Phase 1 Trial With the Cell-Based Immune Primer Ilixadencel, Alone, and Combined With Sorafenib, in Advanced Hepatocellular Carcinoma

**DOI:** 10.3389/fonc.2019.00019

**Published:** 2019-01-21

**Authors:** Magnus Rizell, Malin Sternby Eilard, Mats Andersson, Bengt Andersson, Alex Karlsson-Parra, Peter Suenaert

**Affiliations:** ^1^Transplantation Center, Sahlgrenska University Hospital, Gothenburg, Sweden; ^2^Department of Surgery, Institute of Clinical Sciences, Sahlgrenska Academy, University of Gothenburg, Gothenburg, Sweden; ^3^Department of Radiology, Sahlgrenska University Hospital and Sahlgrenska Academy, University of Gothenburg, Gothenburg, Sweden; ^4^Department of Radiology, Karolinska University Hospital, Huddinge, Sweden; ^5^Department of Microbiology and Immunology, Sahlgrenska University Hospital and Sahlgrenska Academy, University of Gothenburg, Gothenburg, Sweden; ^6^Immunicum AB, Stockholm, Sweden; ^7^Department of Immunology, Genetics and Pathology, Uppsala University, Uppsala, Sweden

**Keywords:** hepatocellular carcinoma, cell therapy, immunotherapy, allogeneic, dendritic cells, ilixadencel, sorafenib

## Abstract

Several lines of evidence support immunotherapy in hepatocellular carcinoma (HCC). We have shown that intratumoral injections of the immune primer ilixadencel (pro-inflammatory allogeneic dendritic cells) are safe in renal-cell carcinoma. Here, we assessed ilixadencel as a single agent and combined with sorafenib in advanced HCC. Of 17 HCC patients enrolled, 12 patients received ilixadencel at the dose of 10 × 10^6^ cells (six as monotherapy and six in combination with sorafenib), and five received ilixadencel at the dose of 20 × 10^6^ cells as monotherapy. The primary objective was to evaluate tolerability. All patients had at least one adverse event, with 30% of such events considered as treatment-related, with one single treatment-related grade three event. The most common toxicity was grade 1 and 2 fever and chills. Eleven of 15 evaluable patients (73%) showed increased frequency of tumor-specific CD8^+^ T cells in peripheral blood. Overall one patient had a partial response (with ilixadencel as monotherapy), and five had stable disease as overall best response per mRECIST. The median time to progression was 5.5 months, and overall survival ranged from 1.6 to 21.4 months. Our study confirms the safety of ilixadencel as single agent or in combination with sorafenib and indicates tumor-specific immunological responses in advanced HCC.

**Clinical Trial Registration:** www.ClinicalTrials.gov, identifier: NCT01974661

## Introduction

Liver cancer, mostly represented by hepatocellular carcinoma (HCC), is the second cause of death from cancer worldwide (1). Although the burden of HCC is heaviest in Asia (2, 3), HCC is the most rapidly increasing cause of death from cancer in the US (4). Likewise, even though the current and projected future epidemic behavior of HCC worldwide is largely conditioned by known and undiagnosed cases of chronic viral hepatitis and alcoholic liver damage, the burden of HCC in Western countries is expected to increase as an end result of the metabolic syndrome and nonalcoholic fatty liver disease (3, 4). Regardless of etiology, however, HCC is the end result of chronic inflammatory injury to the liver.

Developing effective therapy for HCC is a difficult task: more than 70% of patients present with advanced disease that is incurable by surgical resection, ablation or transplantation (5), and implementing treatment for HCC depends not only on tumor extent but also on the level of underlying hepatic dysfunction, present in nearly 90% of cases (6). For patients who are not considered to be curable, locoregional modalities, or systemic therapy may be offered with palliative intent. For nearly a decade, sorafenib has remained the only approved agent leading to gain in overall survival (OS) in first-line palliative therapy (7, 8). Several recent phase 3 trials have yielded negative results in terms of OS improvement (6, 9–11). Recently, however, lenvatinib was shown to be non-inferior to sorafenib for OS in the first-line setting (12). This led to approval in the US by FDA. Additionally, regorafenib and nivolumab have been approved in the US after failure of sorafenib (13, 14). Unfortunately, the median OS of patients with HCC and preserved liver function receiving first-line therapy with sorafenib or lenvatinib is only around 1 year (7, 10, 12, 15). Likewise, patients eventually receiving regorafenib or cabozantinib after failure of sorafenib have an expected median OS close to 10 months (13, 16).

Recent favorable results from the use of the programmed cell death 1 (PD-1) inhibitors nivolumab (14) and pembrolizumab (17) as single agents hopefully herald the expected benefit from immunotherapy in HCC (18). Indeed, several lines of evidence point to a potential role for immunotherapy in HCC, including the viral origin of the disease; its high mutation burden and expression of tumor-associated antigens; the association of immunological findings and prognosis; and the existence of an immunosuppressive environment within tumors (18, 19). The latter phenomenon is due in part to myeloid-derived suppressor cells (MDSCs), a diverse group of myeloid cells capable of suppressing antitumor immunity. This provides a rationale for combining immunotherapy with MDSC inhibitors (20).

Among the several immunotherapy strategies that can be useful against HCC, we have focused on tumor neoantigens as promising targets for intervention. Ilixadencel, which consists of monocyte-derived, allogeneic dendritic cells (DCs) that are stimulated with a combination of pro-inflammatory factors, can be administered by intratumoral injections and function as an immune primer (21, 22). Based on preclinical *in vitro* and *in vivo* data (20, 21, 23) we expect that intratumorally injected pro-inflammatory allogeneic DCs in the clinical setting will induce recruitment of immune cells, including Natural killer (NK) cells, DCs and T cells to the injection site. The cross-talk between the DCs and recruited NK cells will induce NK cell activation, subsequently leading to local tumor-cell killing and release of cell-associated tumor antigens. NK-cell derived interferon-gamma (IFN-γ), in concert with tumor necrosis factor-alpha (TNF-α) produced by the injected DCs and by activated NK cells will enhance cross-presentation of captured tumor antigens by recruited, endogenous, DCs. These antigen-loaded and cross-presenting DCs will start to mature due to activation by pro-inflammatory factors like TNF-α and interleukin (IL)-1β released by the injected allogeneic DCs. Production of IFN-γ by recruited and subsequently activated NK cells and alloreactive T cells will furthermore favor the differentiation of Th1 polarizing DCs. Additionally, NK-cell and alloreactive T cell-derived IFN-γ may inhibit immunosuppressive M2-macrophages (24) and drive Treg fragility within the tumor (25). The approach to inject monocyte-derived DCs intratumorally and thereby use the tumor as the antigen source has previously been tested in the HCC setting (26). However, in the latter study the DCs were autologous and engineered to produce IL-12 by recombinant adenovirus transfection and aimed to pick up tumor-derived antigens within the tumor and present these antigens to tumor-specific T cells. No objective tumor response was observed in the treated HCC patients (*n* = 9), but stable disease as best response was observed in two out of nine HCC patients.

In a first-in-man, dose-escalation trial, intratumoral injections of ilixadencel—in doses ranging from 5 to 20 × 10^6^ viable cells—have been shown to be safe and to lead to immunological responses among patients with metastatic renal-cell carcinoma (20). In the current phase 1 trial, we assessed the safety and activity of ilixadencel as a single agent and combined with sorafenib in the treatment of patients with advanced HCC. In addition to its current role in HCC, sorafenib has been found to inhibit MDSCs and regulatory T cells in a preclinical model of HCC (27). Sorafenib has further been demonstrated to reduce the frequency and expression pattern of immune checkpoint receptors, regulatory T cells, MDSC and the levels of immunosuppressive cytokines in HCC patients (28). These preclinical and clinical data supported the concept of combining sorafenib with ilixadencel. On the other hand, however, preclinical data indicate that sorafenib inhibits LPS and poly-IC induced DC maturation and T cell activation (29).

## Materials and Methods

### Study Design, Oversight, and Objectives

The current trial (NCT01974661) was designed by the sponsor and academic investigators, who vouch for its integrity and the contents of this manuscript. The trial was conducted entirely at Sahlgrenska University Hospital, Gothenburg, Sweden. The study was approved by its institutional review board. All patients provided written informed consent in accordance with the Declaration of Helsinki before entering the trial. A safety committee composed by the principal investigator, two independent physicians, the medical expert at the Sponsor and the project lead at the contract research organization provided oversight during the conduct of the trial, ensuring the safety of dose escalation.

The protocol foresaw inclusion of a maximum of 18 patients. The study enrolled 17 HCC patients. The first 12 patients were treated with ilixadencel as a single agent at two dose levels, with no intra-patient dose escalation. The starting dose of 10 × 10^6^ viable cells was planned for the first six patients, whereas the following six would receive 20 × 10^6^ viable cells if the starting dose did not cause dose-limiting toxicity (DLT) in more than two of six patients. Up to an additional 6 patients were treated with the combination of ilixadencel (at the dose of 10 × 10^6^ viable cells) and approved doses of sorafenib. DLT was defined as any of the following adverse events if they were possibly or probably related or worsened due to study treatment: grade ≥4 thrombocytopenia; a rise of more than three points in Child-Pugh score between days 1 and 8 of each ilixadencel injection; grade ≥4 hyperbilirubinemia; grade ≥3 fever; or any other medically relevant grade ≥3 event. All adverse events were classified according to the Common Terminology Criteria, version 4.03.

The primary objective of this study was to evaluate the tolerability and toxicity of ilixadencel in this setting. Secondary objectives were to assess radiographic, immunological and inflammatory responses to treatment; to describe changes in liver function, Eastern Cooperative Oncology Group (ECOG) performance status, and quality of life scores; and to estimate time to progression and OS. During study conduct, an exploratory objective was added and consisted in the pharmacodynamic assessment of the effect of ilixadencel alone or combined with other agents *in vitro* (see below).

### Preparation of Ilixadencel

The manufacturing of ilixadencel (Immunicum AB, Gothenburg, Sweden) has been described in details previously (20). In brief, manufacturing took place at the Cancer Centre Karolinska, Stockholm, Sweden. Donor screening and donor eligibility were done in accordance with country-specific law and implemented EU directives. More specifically, the donors for the leukapheresis source material used for manufacture of the batches used in the HCC study were tested negative for syphilis, HIV, hepatitis A, B, and C. Monocytes were isolated by elutriation from the leukapheresis source material. In brief, the leukapheresis source material was fractionated by counterflow elutriation in a closed system (ELUTRA®; Terumo BCT, Lakewood, CO, USA). Five (5) fractions were collected in total, all were sampled but only fraction 5 (monocyte fraction) was tested for blood cell composition, cell count and viability. Flow cytometry, using fluorochrome-conjugated antigen-specific antibodies, was used to evaluate cellular composition (monocytes, CD14; T cells, CD3; B cells, CD19; and NK cells, CD56) in the monocyte fraction. The fraction consisted of approximately 95% CD14 positive cells. The monocytes were cultured in CellGro® DC Medium (CellGenix™, Freiburg, Germany), a serum-free cell-culture medium, supplemented with 100 ng/mL granulocyte macrophage colony-stimulating factor (GM-CSF) and 20 ng/mL interleukin (IL) 4 (both cytokines from CellGenix™). Cells were then cultured in a closed system and incubated at 37°C in 5% CO2 atmosphere. After differentiation into immature DCs in medium supplemented with 100 ng/mL of GM-CSF and 20 ng/mL of IL-4, a cocktail of activation/maturation factors consisting of the toll-like receptor (TLR) 7/8 agonist R848 (2.5 μg/mL; Invivo-Gen, San Diego, CA), the TLR3 agonist Poly I:C (20 μg/mL; Sigma-Aldrich, St. Louis, MO) and human recombinant IFN-γ (1000 U/mL; Boehringer-Ingelheim, Ingelheim am Rhein, Germany) was added to the culture medium. After maturation, pro-inflammatory DCs were harvested and resuspended in heat-inactivated AB plasma, and supplemented with 10% dimethyl sulfoxide. Aliquots of the cell suspension were frozen to −150°C at a controlled rate, and the final product was thawed and tested for sterility, mycoplasma, and endotoxin before clinical use. Release criteria also included cell viability, phenotype (HLA-DR and CD86 expression) and function as measured by IL-12p70 and RANTES production after thawing (20). All doses of ilixadencel used in this trial originated from two different batches from the same donor.

### Patient Eligibility, Treatment, and Assessment

Eligible patients were aged 18 years or older and had an ECOG performance status of 0 or 1 and adequate organ function; they also had HCC confirmed by histopathology or non-invasively by European Association for the Study of the Liver criteria (30), and at least one radiologically measurable liver lesion. Initially, eligibility required the presence of stage B and C according to the Barcelona Clinic Liver Cancer system (BCLC) (31) and ineligibility to transarterial chemoembolization (TACE); after treatment of the first 12 patients, the protocol was amended to also allow patients with BCLC stage A, B, or C disease and eligible to TACE or to receive sorafenib (during the trial or having started it no longer than 4 weeks before enrolment). Key exclusion criteria were poor liver function (7 or more points in the Child-Pugh score); active autoimmune disease requiring systemic treatment, use of immunosuppressives within 28 days, or previous organ transplantation; and active hepatitis B or C, or any other infection, requiring treatment. Further details about inclusion and exclusion criteria can be found in the Supplementary (Panel S1).

Three intratumoral injections of ilixadencel were planned for each patient. The first was planned for study day 1, the second was to be administered 14-21 days after the first, and the third was planned for 21-35 days after the second. Each injection was performed under ultrasound guidance and into one hepatic lesion. Patients were instructed to arrive to the clinic in the morning of the day of injection in a fasting state of 4 hours. Injections took place at the Department of Radiology, and patients remained hospitalized at least 18 hours after each injection. Thereafter, patients were assessed for safety within 2 days from the next injection, 28-35 days after the third injection, and at 3 and 6 months. Tumor dimensions were assessed at baseline, at 3 and 6 months after the first injection, and every 3 months until disease progression or the end of the study. Several immunological parameters were assessed, including change from baseline in tumor-specific T cells [after *in-vitro* stimulation with the HCC-associated tumor peptides, alpha-fetoprotein (AFP) and human telomerase reverse transcriptase (hTERT)], systemic levels of selected cytokines, chemokines and other inflammatory mediators, and markers of auto- and alloimmunization. Objective response assessment was done using computed tomography or magnetic resonance imagining and followed the HCC-specific, modified Response Evaluation Criteria in Solid Tumor criteria (30).

### Analysis of Antigen-Specific T lymphocytes

Frozen patient peripheral-blood mononuclear cells (PBMCs) taken before the first dose of ilixadencel and 1 week after the third dose of ilixadencel were analyzed by flow cytometry. Mixes of overlapping peptides for AFP and hTERT were added at a final concentration for each peptide mix of 10 ug/mL. Using overlapping peptides spanning an entire protein sequence, CD8^+^ T-cell responses can thus be detected to multiple epitopes, regardless of HLA type. The peptide mixes were produced by JPT Peptide Technologies GmbH, Berlin, Germany. A CD8 T-cell detection cocktail consisting of a FITC-conjugated antibody against IFN-γ and APC-Cy7-conjugated anti-CD8 was used. The frequency of tumor-specific, IFN-gamma producing, CD8^+^ T cells was obtained by subtracting the percentage of positively stained cells in control samples (tumor peptide mix not added) from the percentage of positively stained cells where the tumor peptide mix was added. A mix of overlapping peptides from CMV, EBV and influenza A viruses was used as positive control. Positive and negative controls were used in the analyses of all pre and post samples.

### Tracking of Injected Ilixadencel Cells

Samples were taken 1 and 24 hrs after each administration of ilixadencel. Flow cytometry analysis for donor cell tracking was performed on isolated PBMCs from peripheral blood samples by staining with selected antibodies that are specific for HLA class I or one HLA-class II antigens and are selectively expressed on donor vaccine cells. Two vaccine cell markers (antibodies) were used, one that stains cells expressing HLA-A2 and one that stains cells expressing HLA-A24 (vaccine cells expressed both HLA-A2 and A24) and read-out was performed by flow cytometry (determining % positive cells out of total number of peripheral blood mononuclear cells). The detection level for vaccine cell tracing was 0,0001% out of total circulating mononuclear cells (monocytes+ lymphocytes) in blood corresponding to 2 million of injected vaccine cells. This process has been described in detail previously (20).

### Inflammatory Immune and Immune Cell Parameters

Cytokines and chemokines were quantified from serum samples taken 30 minutes after each administration of ilixadencel, using a Bio-Plex human cytokine assay (Bio-Rad Laboratories AB, Sundbyberg, Sweden). The analysis was performed according to the manufacturer's instructions. The following inflammatory parameters were analyzed: IL-1R, IL-2, IL-4, IL-5, IL-6, IL-7, IL-8, IL-10, IL-12p70, IL-13, IL-17A, G-CSF, GM-CSF, IFN-gamma, MCP-1, MIP-1 beta and TNF-alpha. Immune cell phenotyping was conducted with standard flow cytometry.

### *In vitro* Studies

One central part of the proposed mode of action for intratumorally injected ilixadencel is to recruit alloreactive CD3^+^ T cells leading to a mixed leukocyte reaction (MLR) which is known to produce factors that induce T helper 1 polarized maturation of “bystander” DCs (22, 32). The impact of potentially co-administrated drugs like sorafenib, anti-PD-1 antibodies or sunitinib on CD3^+^ T cell activation/proliferation against ilixadencel and release of inflammatory cytokines in the MLR was therefore investigated *in vitro*. This exploratory objective was conducted in parallel to the current clinical study but did not involve samples from the accrued patients. The effect of ilixadencel on PBMCs of allogeneic healthy donors was assessed in combination with fixed concentrations of sorafenib tosylate (1 μg/mL), sunitinib malate 0.1 μg/mL, an anti-PD-1 antibody (20 μg/mL), and control mouse immunoglobulin (Ig) G1. In brief, PBMCs from whole blood donated by five healthy volunteers who tested negative for hepatitis B and C viruses, as well as HIV, were used in mixed leukocyte reaction assays. Such assays consisted in co-culture for 5 days of carboxyfluorescein succinimidyl ester-labeled PBMCs from five healthy donors. After 5 days, cells were washed, centrifuged and resuspended in 50 μL phosphate-buffered saline and 0.1% bovine serum albumin for analysis by flow cytometry and of cytokine production. CD3 staining was used to allow identification of the responding T cells. Flow cytometry data were acquired with an iQue® Screener and analyzed using the ForeCyt® 4.1 software (both from IntelliCyt, Albuquerque, NM). Cell-culture supernatants were analyzed for 17 cytokines using Luminex 200 and the xPONENT software (Luminex, Austin, TX).

### Statistical Analysis

All baseline and outcome variables, as well as the pharmacodynamic assays, were analyzed descriptively. Time-to-event endpoints were analyzed using Kaplan-Meier curves.

## Results

### Patient Characteristics and Exposure to Treatment

Between October 2013 and October 2017, 17 HCC patients were enrolled in the trial. The main baseline features of the 17 HCC patients are shown in Table 1. Fifteen patients were men, fourteen had a performance status of 1, and only five had prior surgical resection of HCC.

**Table 1:** Baseline demographic and clinical features.

**Characteristics/Variable**	**10 × 10^**6**^ as single agent**	**20 × 10^**6**^ as single agent**	**10 × 10^**6**^ with sorafenib**	**Total**
Number of patients (%)	6	5	6	17
**AGE (YEARS)**				
<65	2 (33.3)	2 (40)	1 (16.7)	5 (29.4)
≥65	4 (66.7)	3 (60)	5 (83.3)	12 (70.6)
**GENDER**				
Male	4 (66.7)	5 (100)	6 (100)	15 (88.2)
Female	2 (33.3)	0 (0)	0 (0)	2 (11.8)
**ECOG**				
0	1 (16.7)	1 (20)	1 (16.7)	3 (17.6)
1	5 (83.3)	4 (80)	5 (83.3)	14 (82.4)
**PRESENCE OF DISTANT METASTASIS**				
No	3 (50)	2 (40)	2 (33.3)	7 (41.2)
Yes	3 (50)	3 (60)	4 (67.7)	10 (58.8)
**MACROSCOPIC VASCULAR INVASION**				
No	3 (50)	2 (40)	3 (50)	8 (47.1)
Yes	3 (50)	3 (60)	3 (50)	9 (52.9)
**CHILD-PUGH**				
A5	2 (33.3)	1 (20)	0 (0)	3 (17.6)
A6	4 (66.7)	4 (80)	6 (100)	14 (82.3)
**ALPHA-FETOPROTEIN LEVEL**				
<200 μg/L	2 (33.3)	3 (60)	2 (33.3)	7 (41.2)
≥200 μg/L	4 (66.7)	2 (40)	4 (67.7)	10 (58.8)
**PRIOR TREATMENT**				
Systemic	5 (83.3)	2 (40)	0 (0)	7 (41.2)
Locoregional	3 (50)	4 (80)	0 (0)	7 (41.2)

Of the seventeen patients enrolled, as shown in Table 2, the first six received ilixadencel alone at the dose of 10 × 10^6^ viable cells (five as second-line monotherapy and one as first-line monotherapy), five received ilixadencel alone at the dose of 20 × 10^6^ viable cells (three as first-line and two as second-line monotherapy) and six patients received ilixadencel at the dose of 10 × 10^6^ viable cells combined with sorafenib (first-line therapy). Thirteen patients received all three administrations of ilixadencel, two patients received only the first two doses, and two received only the first dose. The reasons for not receiving all three doses were toxicity (*N* = 3) and disease progression (*N* = 1). One of the three such patients with toxicity developed chills after the first dose of 20 x 10^6^ viable cells (as monotherapy) and received 10 × 10^6^ viable cells as subsequent dose; this was the only case of dose reduction. Two patients with toxicity leading to treatment interruption after the first dose had been treated with the combination of ilixadencel and sorafenib.

**Table 2:** Ilixadencel treatment line with respect to dose.

**Characteristics/variable**	**10 × 10^**6**^ as single agent**	**20 × 10^**6**^ as single agent**	**10 × 10^**6**^ with sorafenib**	**Total**
Number of patients	6	5	6	17
**ILIXADENCEL TREATMENT LINE**				
First line	1	3	6	10
Second line	5	2	0	7

### Treatment Safety and Dose-Limiting Toxicity

All patients had at least one adverse event, for a total of 217 events reported after treatment initiation. Thirty-four (16%) of the 217 adverse events were of grade 3 or higher. Sixty-six (30%) of the 217 adverse events were considered by investigators as treatment-related (i.e., at least possibly related to ilixadencel) and 3 of these events were classified as serious treatment-related adverse events (in two patients). One of these serious adverse events were fever episodes of grade 2 that led to prolongation of hospital stay, whereas one episode of fever was accompanied by suspected, unconfirmed sepsis of grade 3. At data cut-off, three patients were still alive, and no deaths were attributed to treatment toxicity.

Table 3 displays the profile of treatment-related adverse events overall and according to dose level and use of sorafenib. The most common toxicity types were fever and chills. There was a single treatment-related grade 3 adverse event, in a patient treated with ilixadencel combined with sorafenib (the case of unconfirmed sepsis), and all other events were grade 1 or 2. There was no DLT as defined in this study. With regard to the time of occurrence of treatment-related adverse events, all events occurred during or shortly after the administration of ilixadencel, and all were reversible. Thirty-six events (in 11 of 17 treated patients) were registered after the first administration of ilixadencel, 20 (in 10 of 16 treated patients) after the second administration, and 10 events were registered (in 5 of 14 patients) after the third injection. Nine of 14 (64%) evaluable patients developed donor-specific alloantibodies, with no apparent relationship with the degree of HLA-mismatch or the dose of ilixadencel (data not shown). No patient developed evidence of induced autoantibody production in response to ilixadencel. Likewise, there was no clear evidence of complement-activation induced by treatment.

**Table 3:** Numbers of patients with treatment-related adverse events, including laboratory abnormalities.

**Treatment-related Adverse Events (AEs)**	**Dose of ilixadencel**			
	All^*^ (*N* = 17)	**10 × 10^**6**^ as single agent (*N* = 6)**	**20 × 10^**6**^ as single agent (*N* = 5)**	**10 × 10^**6**^ with sorafenib (*N* = 6)**
Total number of patients with AEs	15	6	5	4
**GRADE 1 OR 2**				
Pyrexia	8	2	4	2
Chills	8	1	5	2
Hypertension	3	–	1	2
Leukocytosis	2	–	1	1
Thrombocytosis	2	1	–	1
Pain in injection site	2	1	1	–
Increased C-reactive protein	2	2	–	–
Fatigue	2	1	1	–
Headache	2	–	1	1
Nausea	2	1	1	–
Oral mucosal blistering	1	1	–	–
Tachycardia	2	–	1	1
Vomiting	2	1	1	–
Abdominal pain upper	1	–	1	–
Abdominal wall hematoma	1	1	–	–
Increased alanine aminotransferase	1	–	1	–
Anemia	1	1	–	–
Aphthous ulcer	1	1	–	–
Increased aspartate aminotransferase	1	–	1	–
Back pain	1	1	–	–
Increased lactate dehydrogenase	1	–	1	–
Cancer pain	1	1	–	–
Increased hepatic enzyme	1	1	–	–
Non-cardiac chest pain	1	–	–	1
Musculoskeletal pain	1	–	–	1
Pruritus	1	–	1	–
**GRADE 3**				
Sepsis	1	–	–	1

* *All 17 HCC patients had at least one adverse event. Fifteen of seventeen patients displayed a treatment-related event*.

### Responses to Treatment

Eleven of 15 (73%) evaluable patients (pre-samples and/or post-samples were missing from two patients) showed increased numbers of tumor-specific (AFP and/or hTERT) CD8^+^ T cells producing IFN-γ post-treatment when compared with baseline levels (Table 4). The post-treatment measurement was conducted on blood samples collected 1 week after the third dose in 13 patients, 1 week after the second and final dose in one patient and 1 week after the first out of two doses in one patient where the blood sample after the final second dose was missing. Nine out of 11 (82%) evaluable patients receiving ilixadencel alone and 2 out of 4 (50%) evaluable patients receiving ilixadencel in combination with sorafenib showed increased tumor-specific CD8^+^ T cells post-treatment.

**Table 4:** Percentage of tumor-specific (AFP and/or hTERT) CD8^+^ T cells in peripheral blood pre- and post-treatment.

**Dose of ilixadencel**	**Number of doses**	**Treatment line**	**AFP-specific (%)**		**hTERT-specific (%)**	
			**Pre**	Post^*^	**Pre**	Post^*^
10 × 10^6^ as single agent (*N* = 6)	3	2nd	0.7	**1.2**	0.2	**0.5**
	3	2nd	0.2	0.2	0.4	0.2
	3	2nd	0.0	0.0	0.0	**0.1**
	3	2nd	0.0	**0.1**	0.0	**0.5**
	3	2nd	0.0	0.0	0.0	0.0
	2	1st	0.4	1.1^**^	0.1	0.0^**^
20 × 10^6^ as single agent (*N* = 5)	3	1st	0.0	0.0	0.0	**0.5**
	2	1st	0.2	**0.4**	0.1	0.1
	3	1st	0.2	**0.3**	0.4	0.3
	3	2nd	0.7	**1.1**	0.0	0.0
	3	2nd	0.4	**0.6**	0.2	**0.5**
10 × 10^6^ with sorafenib (*N* = 6)	3	1st	0.0	0.0	0.2	0.0
	3	1st	0.0	0.0	0.0	0.0
	3	1st	0.0	**0.2**	0.7	0.4
	3	1st	0.1	0.1	0.0	**0.1**
	1	1st	ND	ND	ND	ND
	1	1st	ND	ND	ND	ND

* *One week after the final ilixadencel dose*.

** *Post-data only available after the first out of 2 doses. Bold figures indicate increase of tumor-specific CD8^+^ T cells post-treatment. Not determined; ND*.

None of the cytokines, chemokines or other inflammatory mediators measured in peripheral blood displayed significant changes with treatment (data not shown).

Radiographic tumor responses could be assessed in 14 of the 17 HCC patients. The three patients without radiographic evaluation evolved to clinical progression before the planned assessment at 3 months and died on study. With respect to the overall best response rate as per modified RECIST during the study, 1 patient out of 17 (6%) had a partial response, 5 patients (29%) displayed stable disease, 7 patients were progressing and 1 patient was considered non-evaluable (1/17 or 6%). Taking the three patients with clinical progressive disease without imaging follow-up into account gives a total of 10 patients with progressive disease (10/17 or 59%). A waterfall plot with overall best responses as per modified RECIST criteria is shown in the Supplementary Figure S1). As shown in Supplementary Figure S2B, the median time to progression was 5.5 months.

### Dose-Response Relationships

A potential association between the two dose levels of ilixadencel and safety as well as efficacy parameters were explored informally. With regard to safety, 39 of the 66 treatment-related events occurred in 10 patients treated with 10 × 10^6^ viable cells, and the remaining 27 events occurred in five patients treated with 20 × 10^6^ viable cells.

Considering only single-agent ilixadencel, six patients received the dose of 10 × 10^6^ viable cells; five of these patients were in their second line, and one received treatment in the first line. There were 21 treatment-related adverse events in these patients; in one case, the adverse event (pyrexia) was considered serious. Five patients with HCC received the dose of 20 × 10^6^ viable cells. In this subgroup, there were 27 treatment-related adverse events. None of the patients treated with dose of 20 × 10^6^ viable cells displayed any serious treatment-related AE.

Dose and CD8^+^ T-cells: Among the six treated patients with 10 × 10^6^ cell dose as only treatment, four subjects had an increase in tumor-specific CD8^+^ T-cells (against either AFP or hTERT). The latter was also the case in all five patients receiving 20 × 10^6^ dose of ilixadencel as monotherapy and in 2 out of 4 evaluable patients treated with the combination of ilixadencel and sorafenib.

Dose and overall best response: In the 20 × 10^6^ dosing group of five patients, one patient had a partial response and one displayed a stable disease as overall best response while the remaining three patients only progressed. Among the patients who received monotherapy ilixadencel at 10 × 10^6^ dose, three had stable disease and three had progressive disease. In the combination treatment group (sorafenib + ilixadencel) of six patients, one patient had stable disease, four progressive disease and one was not evaluable.

### Overall Survival, Liver Function, Performance Status, and Quality of Life

As of 30 August 2017, the median time to progression among the 17 HCC patients was 5.5 months [see Supplementary Material for the Kaplan-Meier curves Supplementary Figure S2 for OS (Panel A) and time to progression (Panel B)]. Likewise, OS ranged from 1.6 to 21.4 months, with three patients still alive at the cut-off date. The Kaplan-Meier median OS times were 7.5 months overall, 7.4 months for the dose of 10 × 10^6^ viable cells (*N* = 12), and 11.8 months for the dose of 20 × 10^6^ viable cells (*N* = 5). Taking treatment line and combination with sorafenib into account, the median OS was 2.7 months for single-agent ilixadencel in the first line (*N* = 4), 10.9 months for single-agent ilixadencel in the second line (*N* = 7), and 8.2 months for patients treated with the combination (*N* = 6). Analyses of liver function, performance status, and quality of life showed that ilixadencel did not influence the natural evolution of these parameters.

### Inflammatory Immune and Immune Cell Parameters

None of the evaluated inflammatory parameters (IL-1R, IL-2, IL-4, IL-5, IL-6, IL-7, IL-8, IL-10, IL-12p70, IL-13, IL-17A, G-CSF, GM-CSF, IFN-gamma, MCP-1, MIP-1 beta and TNF-alpha) or immune cell parameters (number of circulating CD3+, CD3+4+ and CD3+8+ T cells, CD19+ B-cells CD3-16+56+ NK cells, CD3-16+56+69+ activated NK cells, CD3+16+56+ NKT-cells, CD3+16+56+69+ activated NKT-cells and CD3+HLA-DR+ activated T cells) exhibited any significant increase or decrease after administration of ilixadencel (data not shown).

### Pharmacokinetic Measures

Ilixadencel cells were detectable in peripheral blood in a total of 6 of the 18 patients: a measurable increase, corresponding to 21–76% of injected cells, was observed in the circulation 1 h after administration in 4 patients, and corresponding to 20–30% of injected cells 18–24 h after administration in 3 of the 4 latter patients and in 2 other patients. No correlation between these cases of blood-seeding of intratumorally injected cells and clinical symptoms like fever, chills or other immune parameters was observed.

### *In vitro* Measures

**Figure 1:**
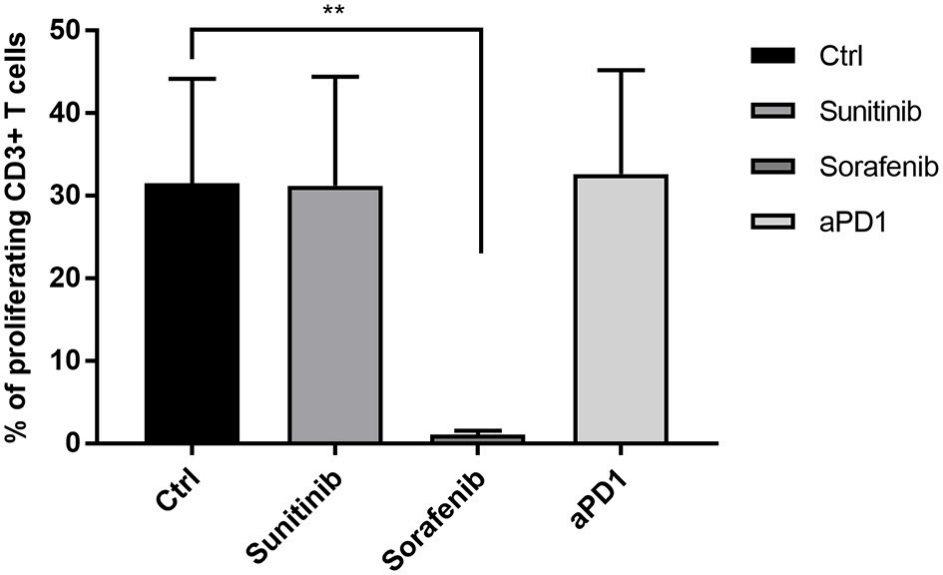
T-cell activation in mixed leukocyte reaction with allogeneic PBMCs and ilixadencel. CD3^+^ T-cell proliferation after 5 days in a mixed leukocyte reaction with allogeneic PBMCs and in the presence of ilixadencel. The effect of ilixadencel on PBMCs from five different donors was assessed in combination with sunitinib (0.1 μg/mL), sorafenib (1 μg/mL) or anti-PD-1 antibody (20 μg/mL; and defined as anti-PD1 in bar), and compared to control (Ctrl), containing ilixadencel and allogeneic PBMCs. CD3 staining was used to allow identification of the responding proliferating T cells by flow cytometry. Results are shown as the percentage of CD3^+^ T proliferating cells and each bar represents mean ± standard deviation (*n* = 5). Statistical significant differences were analyzed using Student's *t*-test. ^**^P < 0.01.

As shown in Figure 1, the addition of anti-PD1 or sunitinib to the ilixadencel/PBMC MLR did not significantly affect T-cell proliferation. The addition of anti-PD1 or sunitinib to the ilixadencel/PBMC MLR did not significantly change cytokine production, except for IL-1 beta and IL-2, whose production was increased in all five ilixadencel/PBMC MLRs after addition of anti-PD1 (see Supplementary Figure S3). However, addition of sorafenib markedly inhibited the proliferative response (Figure 1) and production of the majority of the cytokines tested in all MLRs (data not shown).

## Discussion

This phase 1 trial, the second with ilixadencel as a single agent, confirms the safety of using doses of 10 and 20 × 10^6^ viable cells of this product for up to three intratumoral administrations, and provides initial evidence that monotherapy and its combination with sorafenib is safe (at the dose of 10 × 10^6^ viable cells) among patients with advanced HCC. Of note, the majority of our patients displayed baseline disease characteristics that reflect a high disease burden. The toxicity profile observed in the current study mirrors the experience in the phase 1 trial among patients with renal-cell carcinoma, in which fever and chills predominated as the treatment-related adverse events and no DLT was found in doses up to 20 × 10^6^ viable cells (20). Likewise, no evidence of autoimmunity was found in either this or the previous trial. As to the frequently observed fever reactions after administration of ilixadencel, we believe that this reaction is mainly induced by the inflammatory process created by the injected cells, actively secreting pro-inflammatory mediators and the subsequent activation of locally recruited immune cells (including NK cells) from the patient. It could be argued that the inflammation caused by physical mechanism in relation to tumor puncture may induce a fever reaction, however published data indicates that the frequency of fever reactions due to liver biopsy are >1% (33).

Similarly to the RCC study (20), there was evidence of a tumor-specific immune response to vaccination with ilixadencel (as well as ilixadencel-specific humoral alloimmunization). A substantial proportion of HCC patients developed ilixadencel-specific alloantibodies, which is in line with the central role for “passenger” allogeneic DCs within transplanted organs as the main inducers of T-cell dependent immunization against donor-derived, non-self, HLA class I and class II antigens (34). This immunization process reflects the cross-presentation of immunogenic donor-derived HLA epitopes by recipient DCs to CD4^+^ T cells, also called the “indirect pathway of allorecognition,” that subsequently promote help to allo-specific B cell clones to differentiate into mature plasma cells producing allo-specific IgG antibodies. It is therefore tempting to speculate that phagocytosis and subsequent concomitant cross-presentation of ilixadencel-derived allogeneic HLA peptides on HLA class II and tumor-associated peptides on HLA class I by recipient DCs will occur, and that the alloreactive CD4^+^ T cells will function as helper cells also for tumor-specific CD8^+^ T cells. In line with such a hypothesis, the majority of HCC patients treated with ilixadencel exhibited an increased frequency of tumor-specific CD8^+^ T cells in peripheral blood. These tumor-specific CD8^+^ T cells were furthermore producing IFN-γ upon *in vitro* stimulation, indicating a cytolytic potential. On the other hand, it could be argued that the development of HLA-antibodies may inhibit the MLR during a second/third administration of ilixadencel from the same donor due to masking of allogeneic HLA molecules on the injected ilixadencel DCs. However, if this is the case *in vivo*, a NK cell mediated Fc-receptor-dependent activation and subsequent release of inflammatory factors (35) may compensate for such potential negative impact on the inflammatory response by HLA-antibodies.

With regard to a dose-response relationship for ilixadencel monotherapy, safety, as well as immunologic and clinical activity, did not differ appreciably between the doses of 10 and 20 × 10^6^ viable cells, administered to six and five patients, respectively. Based upon these results, it seems early to draw definitive conclusions about the dose for future development, and further research is warranted. Of note, a clear dose-response relationship is not always found with DC vaccination, although a significant association has been found between the dose of DC-based vaccines and the clinical benefit rate in prostate cancer and RCC (36).

With regard to OS in the different subgroups, the subgroup of HCC patients treated with ilixadencel in combination with sorafenib (*N* = 6), a kinase inhibitor that resembles sunitinib in its ability to inhibit MDSCs (27), had a median OS that was shorter than expected when compared with historical controls receiving first-line sorafenib. Notably, data from our *in-vitro* studies clearly indicated that sorafenib, in contrast to sunitinib or anti-PD-1 antibodies, markedly inhibits the MLR between ilixadencel and allogeneic PBMCs. Although induction of tumor-specific T cells was less frequent and median OS shorter among patients treated with ilixadencel in combination with sorafenib as first-line therapy (*N* = 6) (50% and 8.6 months, respectively) than among those who received ilixadencel as monotherapy in the second-line setting (*N* = 7) (78% and 10.9 months, respectively), the small sample size precludes definitive conclusions about a potential inhibition of ilixadencel-dependent activation of tumor-specific CD8^+^ T cells *in vivo* and subsequent clinical efficacy. The very short median OS for the 4 patients receiving ilixadencel as first line monotherapy may at least by part be explained by a relatively poor performance status at inclusion in 3 out of 4 patients (ECOG 1 and Child-Pugh A6). These three patients also had AFP-levels >200 μg/L and distant metastasis, and two of these 3 patients also had macrovascular invasion. Notably, the only patient in this first-line subgroup who had a normal performance status (ECG 0), Child-Pugh A5 and AFP level below 200 ug/L, survived for 19 months despite macrovascular invasion at diagnosis.

Taken together, the current results provide evidence that intratumoral administration of ilixadencel induce a tumor-specific immune activation in a substantial number of patients and thus support the strategy of further developing ilixadencel as a tumor-specific immune primer, particularly in combination with drugs known to inhibit tumor-derived immunosuppression like sunitinib and gemcitabine, as well as checkpoint inhibitors. In the case of sorafenib, which also is known to inhibit tumor-derived immunosuppressive mechanisms in mice and humans (27, 28), the concomitant negative impact on DC-maturation and DC-mediated T cell activation (29) may however, lead to a negative net effect, as indicated in the present study. Through its ability to prime the immune system, and given its safe toxicity profile, ilixadencel can be tested as a component of immunotherapy in most types of solid tumors.

## Data Availability Statement

Immunicum AB is a public company listed on the Nasdaq, Stockholm, Sweden. The datasets used and/or analyzed during the current study are available from the corresponding author on reasonable request and when a confidentiality agreement including a clause about compliance to securities law have been signed.

## Ethics Statement

The trial was conducted entirely at Sahlgrenska University Hospital, Gothenburg, Sweden. The Ethics Review Board is Regionala etikprövningsnämnden i Göteborg with postaddress: Box 401, 405 30 Göteborg, Sweden. The protocol, the patient information and the informed consent form were reviewed and approved by the Ethics Review Board (ERB) in Gothenburg (approved 14th of August 2013) and by the Swedish Medical Products Agency (approved 20th of June 2013), before study start. The study was conducted in compliance with the study protocol and in accordance with the International Conference in Harmonization–Good Clinical Practice guidelines (ICH-GCP), the principles of the Declaration of Helsinki and applicable local regulatory regulations and directives for clinical trials. It was the responsibility of the Investigator to provide each patient with full and adequate verbal and written information about the objectives, procedures and possible risks and benefits of the study as well as information on data handling, storage of biological samples, the patient's rights according to the Law on Personal Data and the Act, Biobanks in Medical Care, access to the medical records for representatives from Sponsor/CRO/Regulatory Authorities, and of their freedom to withdraw from the study at any time without needing to give any particular reason. All patients were given the opportunity to ask questions about the study. The informed consent was signed by the patient and the Investigator before any study specific procedures started.

## Author Contributions

MR and MS designed the study and assisted with provision of patients and performed data analyses. MA performed all image-guided intratumoral injections of the drug product. BA performed data analyses. AK-P and PS designed the study, performed data analyses and drafted the manuscript. All authors were involved in manuscript preparation and approval of final version.

### Conflict of Interest Statement

BA, AK-P and PS report ownership of stocks in Immunicum AB. AK-P and PS are Immunicum AB employees. The remaining authors declare that the research was conducted in the absence of any commercial or financial relationships that could be construed as a potential conflict of interest.

## Abbreviations

AFP: alpha-fetoprotein
BCLC: Barcelona Clinic Liver Cancer system
DCs: dendritic cells
DLT: dose-limiting toxicity
ECOG: Eastern Cooperative Oncology Group
GM-CSF: granulocyte macrophage colony-stimulating factor
HCC: hepatocellular carcinoma
hTERT: human telomerase reverse transcriptase
IFN-γ: interferon-gamma
IL: interleukin
MDSCs: myeloid-derived suppressor cells
MLR: mixed leukocyte reaction
NK: natural killer
OS: overall survival
PBMCs: peripheral-blood mononuclear cells
PD-1: programmed cell death 1
TACE: transarterial chemoembolization
TNF-α: tumor necrosis factor-alpha.
