# Supplementary material for: Phase 1 Trial With the Cell-Based Immune Primer Ilixadencel, Alone, and Combined With Sorafenib, in Advanced Hepatocellular Carcinoma
Source: Front Oncol. 2019 Jan 21;9:19. doi: 10.3389/fonc.2019.00019 (PMC6348253; doi:10.3389/fonc.2019.00019)
Supplement: Supplementary file 1 [file Data_Sheet_1.docx]

Supplementary Material

Phase 1 Trial with the Cell-Based Immune Primer Ilixadencel, Alone and Combined with Sorafenib, in Advanced Hepatocellular Carcinoma

Magnus Rizell^1,2^, Malin Sternby Eilard^1,2^, Mats Andersson^3,4^, Bengt Andersson^5^,

Alex Karlsson-Parra^6,7^, and Peter Suenaert^6*^

*** Correspondence:** Peter Suenaert, MD, PhD; [peter.suenaert@immunicum.com](mailto:peter.suenaert@immunicum.com)

# Supplementary Panel S1. Complete selection criteria for the trial (NCT01974661)

**Inclusion Criteria**

1 Be informed of the nature of the study and have provided written informed consent.

2 At least 18 years of age.

3 Diagnosis of hepatocellular carcinoma according to European Association for the Study of the Liver criteria or pathology.

4 Radiologically measurable liver tumor(s), i.e at least 20 mm in longest uni-dimensional diameter as measured by CT/MRI.

5 Not eligible for curatively aiming treatment or transarterial chemoembolization (TACE). Tumor stage B or C according to the Barcelona Clinic Liver Cancer.

6 Amendment 3: Tumor stage A, B or C according to the Barcelona Clinic Liver Cancer classification and eligible for sorafenib treatment or having ongoing sorafenib treatment for not more than 4 weeks at the time for inclusion OR eligible for TACE or having received not more than 1 previous TACE treatment.

**Exclusion Criteria**

1 Performance status > Eastern Cooperative Oncology Group 2.

2 Liver function according to Child-Pugh >7 points.

3 Known major reaction/adverse event in connection with previously made vaccination (e.g. asthma, anaphylaxia or other serious reaction).

4 Known major reaction/adverse event in connection with previous transfusions of blood products.

5 Active autoimmune disease requiring treatment with systemic immunosuppressive agents, e.g. inflammatory bowel disease, multiple sclerosis, sarcoidosis, psoriasis, autoimmune hemolytic anemia, rheumatoid arthritis, systemic lupus erythematosus, vasculitis, Sjögren's syndrome, scleroderma, autoimmune hepatitis, and other rheumatological diseases.

6 Tested positive for HIV.

7 Active virus disease (hepatitis B virus and hepatitis C virus) requiring antiviral treatment.

8 Ongoing infection that requires treatment with antibiotics or antiviral medication.

9 Immunosuppression (within 28 days) prior to the first injection of ilixadencel. Inhaled, intranasal and local steroids accepted.

10 Patients with prior history of malignancy other than hepatocellular carcinoma, within the preceding 3 years OR with relapse after complete response, except for 5 years follow-up of adequately treated in situ carcinoma without recurrence or non-melanoma skin cancer.

11 Inadequate laboratory parameters, i.e: a. P-Prothrombin complex >1.4; b. Platelet count <75 x109/L; c. Leukocyte count <3.0 x 109/L; d. P-APT time outside normal limit.

12 Previous organ transplantation.

13 Women of Childbearing Potential refusing to use adequate contraception (oral or injectable contraceptives, hormone releasing intrauterine device) throughout the study period.

14 Pregnant or lactating women.

15 Life expectancy less than 3 months.

16 Anti-tumor treatment (within 28 days) prior to the first injection of ilixadencel, except sorafenib or TACE for patients included according to Amendment 3.

17 For patients included according to Amendment 3: Previous systemic anti-cancer treatment.

18 Investigational treatment (within 28 days) prior to the first injection of ilixadencel.

19 Known blood dyscrasia (bleeding complication),

20 Known malignancy in central nervous system.

21 Any reason that, in the opinion of the investigator, contraindicates that the patient participates in the study.

# Supplementary Figures


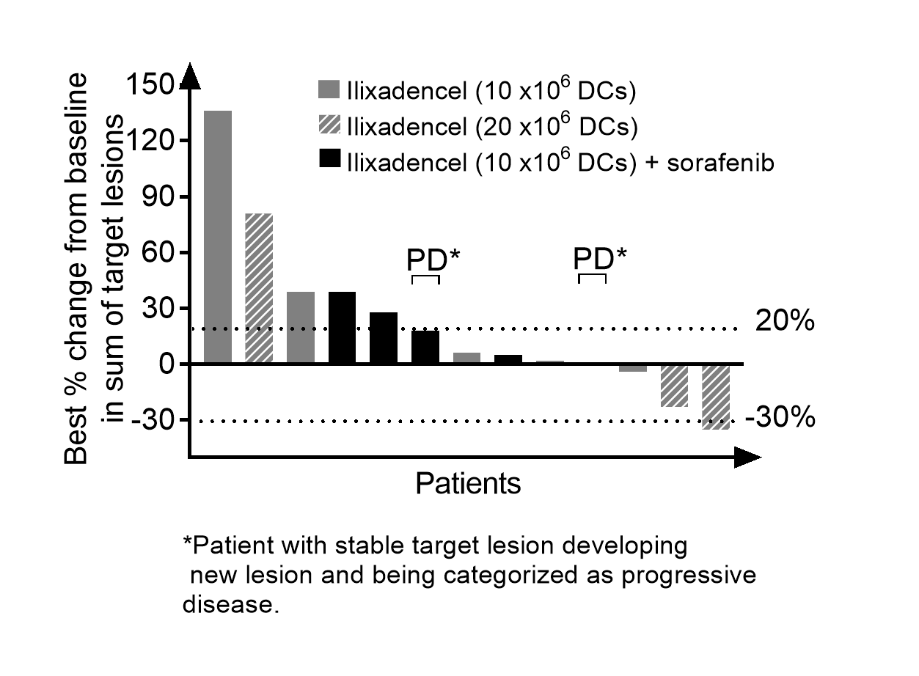


**Supplementary Figure S1. Waterfall plot with change in target-lesion dimensions.** Tumor measurement was done in 14 of 17 patients (3 HCC patients clinically progressed before first re-evaluation) evaluable by CT/MRI at baseline, 3 months and 6 months after the first vaccination, and then every 3 months or until disease progression using the mRECIST criteria. One of the 14 patients was not evaluable for mRECIST and is not represented in the figure. Treatment with respect to doses indicated in different colours and patterns.

**Panel A: Overall survival**


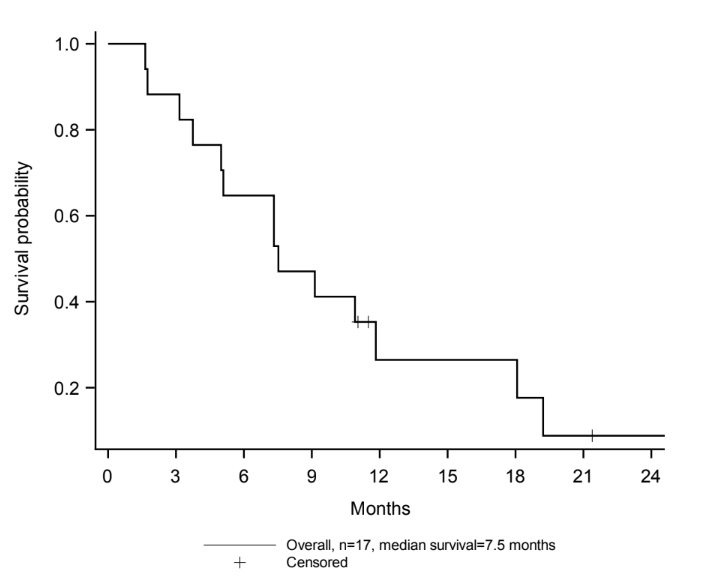


**Panel B: Time to progression**


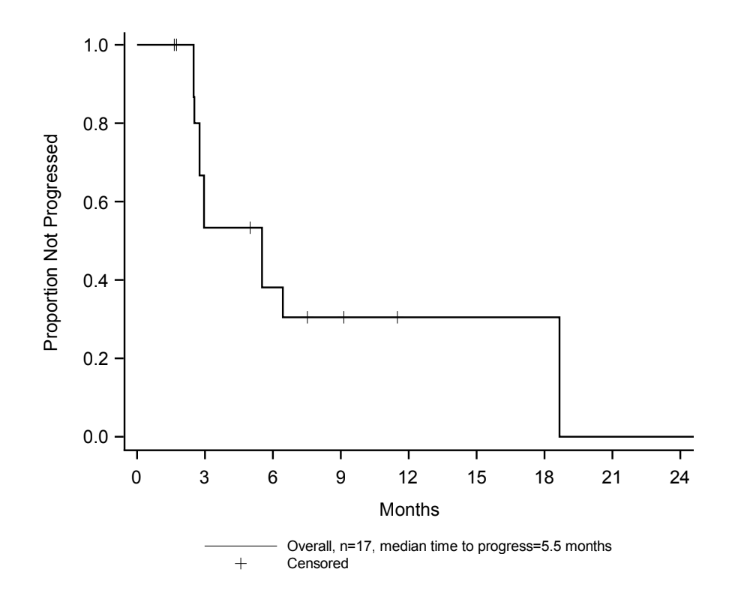


**Supplementary Figure S2. Kaplan-Meier curves for patients with hepatocellular carcinoma (N=17).** Overall survival (OS) (**Panel A**), measured as number of days from first dose of ilixadencel, and time to progression (TTP) (**Panel B**), measured as number of days from first dose of ilixadencel until radiologically proven progression according to mRECIST for all the patients with hepatocellular carcinoma are presented as Kaplan-Meier curves together with the respective median values.


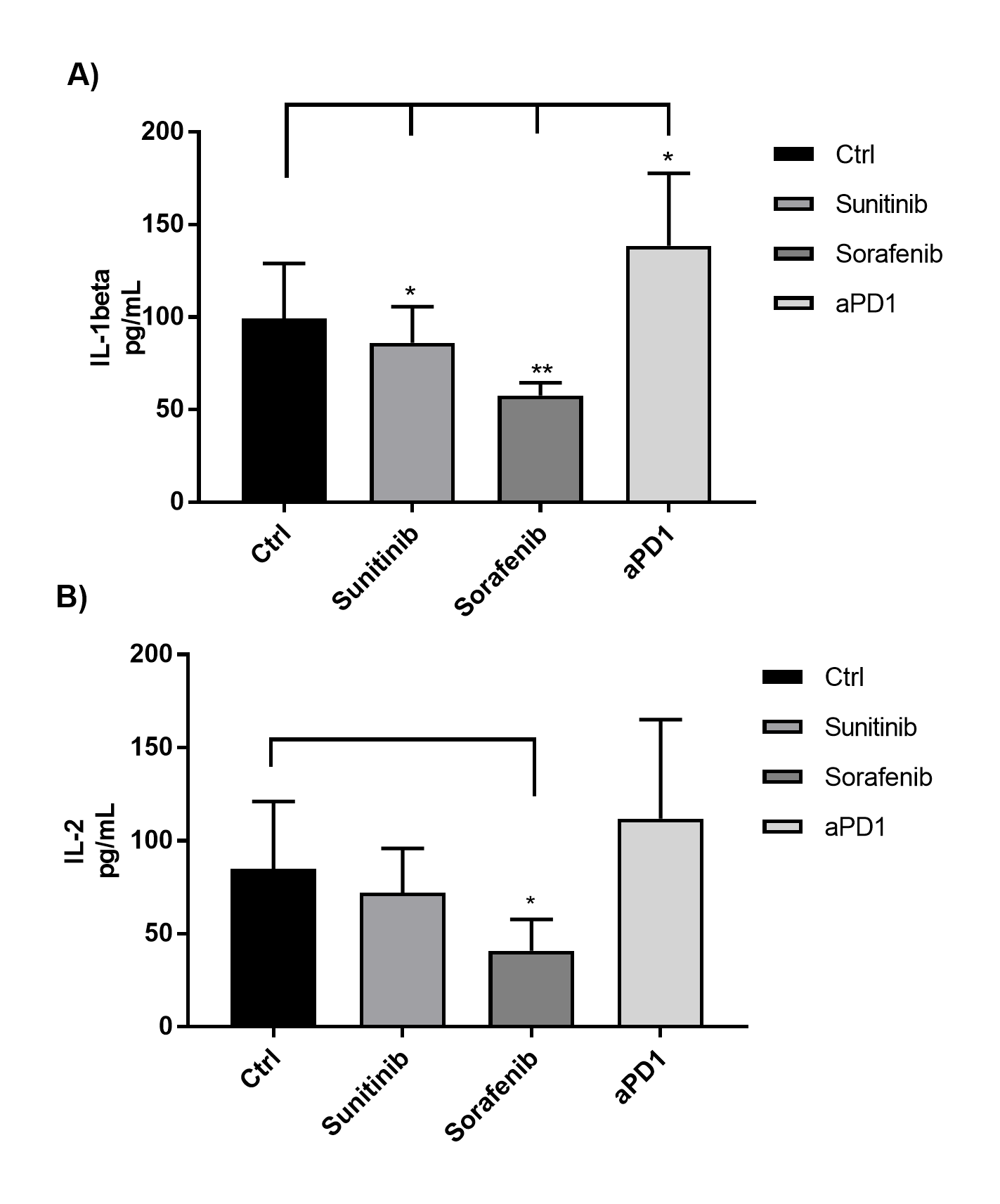


**Supplementary Figure S3. Cytokine production in the mixed leukocyte reaction.** Cytokine production in supernatants harvested after 5 days of co-culture between ilixadencel and allogeneic PBMCs (mixed leukocyte reaction) from five different donors. The effect of ilixadencel on allogeneic PBMCs was assessed in combination with sunitinib (0.1μg/mL), sorafenib (1 μg/mL) or anti-PD-1 antibody (aPD1; 20 μg/mL), and compared to control (Ctrl), containing ilixadencel and allogeneic PBMCs. Supernatants were harvested from cultured cells and analyzed for the production of (**A**) IL-1b and (**B**) IL-2 using a multiplex analyzer. Bars represent mean ± standard deviation (n=5) and signiﬁcance levels, calculated by Student´s t-test, were accepted when *P < 0.05, and **P < 0.01.
